# Consumers preferences on nutritional attributes of dairy‐alternative beverages: hedonic pricing models

**DOI:** 10.1002/fsn3.1757

**Published:** 2020-08-24

**Authors:** Tingyi Yang, Senarath Dharmasena

**Affiliations:** ^1^ International Business School Shaanxi Normal University Xi'an P.R. China; ^2^ Department of Agricultural Economics Texas A&M University College Station TX USA

**Keywords:** consumer preferences, dairy alternative beverages, hedonic pricing models, Nielsen Homescan data, qualitative characteristics

## Abstract

Dairy products, especially milk play a crucial role in assuring dietary quality for U.S. households. However, due to taste, nutrition, health and environmental concerns, households increasingly prefer to consume dairy alternative beverages instead of conventional milk in the U.S. This work is motivated by the need to take into consideration of intrinsic characteristics and differences of such characteristics when analyzing the changes of consumers' purchasing behavior of and willingness to pay for dairy alternative beverages and conventional milk products. After aggregating and organizing the purchase data of Nielsen Homescan and first‐hand nutrition data, this study estimates both linear and semi‐log hedonic pricing models. The results show that consumers exert the highest weights and assign highest evaluation on such qualitative characteristic as nutritional attributes which include calories, protein, fat, vitamin A and vitamin D in which protein is the most valued attribute and other characteristics such as package size, multi pack and brand. The hedonic pricing order and value of these qualitative characteristic are indicative of consumers' purchasing behavior and thus provide essential information for manufacturers to better differentiated their products and develop products catering to consumer's preferred attributes.

## Graphical abstract

Because of the taste, nutrition, health, and environmental concerns, households increasingly prefer to consume dairy alternative beverages instead of conventional milk in the U.S. This work is motivated by the need to take into consideration of intrinsic characteristics and difference of such characteristics when analyzing the changes of consumers' purchasing behavior of and willingness to pay for dairy alternative beverages and conventional milk products. Both linear and semi‐log hedonic pricing models are estimated, and the results show that consumers exert the highest weights and assign highest evaluation on such qualitative characteristic as nutritional attributes which include calories, protein, fat vitamin A and vitamin D, and other characteristics such as package size, multi‐pack, and brand.

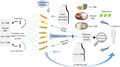

## 1 INTRODUCTION

In the past decade, dairy alternative beverages have gained its market position as a robust competitor for conventional milk in the United States. Consumers have gradually turned away from conventional milk, leading the push towards plant‐based milk products because a growing number of consumers beginning to believe that plant‐based foods are healthier and more environmentally friendly than animal‐based foods. As indicated by Singhal, Baker, and Baker (2017), the increasing sales trend of non‐dairy beverages in westernized counties is due to consideration that foods labeled as natural are perceived to be the most healthy and appropriate nutritional choice by most consumers. Decloedt, Landschoot, Watson, Vanderputten, and Vanhaecke (2017) suggested that to address the ever‐growing group of health‐conscious consumers, more and more nutritional and health claims are being used on food products. Davis, Dong, Blayney, and Owens (2010) and Copeland and Dharmasena (2016) showed that per capita consumption of fluid milk in the United States has been dwindling over the past 25 years. While according to Allied Market Research, the global dairy alternative beverage market is expected to garner $21.7 billion by 2022, registering a compound annual growth rate (CAGR) of 13.3 percent during the period 2016–2022. Plant‐based beverages can be generally classified in five categories: cereal‐based (oat, rice, corn, spelled); legumes‐based (soy, peanut, lupin, cowpea); nut‐based (almond, coconut, hazelnut, sunflower); pseudocereals‐based (quinoa, teff, amaranth; Verduci et al., 2019). One of the main characteristics of these products is that even though they are not real milk products with animal origin, they are often fortified with certain nutrients such as protein, vitamin, and calcium to make them comparable with conventional milk. As is shown in Copeland and Dharmasena (2016), these beverages are designed not only to quench the thirst but also to provide numerous vitamins, minerals, proteins, and favorable fatty acids.

Soy milk and almond milk though are the leading categories in U.S. dairy alternative beverage market, they also face heavy competition from other dairy alternative beverage categories due to the availability of various flavors and tastes and fortification with multiple nutrients. And these milk alternatives vary in their composition of macro and micronutrients (Claeys et al., 2014). As indicated by Aggarwal, Rehm, Monsivais, and Drewnowski (2016), concerns with taste, nutrition, cost, and convenience are said to be key influences on food choices. They also found that taste and nutrition tend to be the most valued attributes among US adults during food shopping. It is because the variety of dairy alternative beverages are different from one another in terms of nutritional composition and price that consumers' relative evaluations of these beverages with different nutritional content and pricing is of great importance to manufacturing and marketing these products. However, the nutritional composition of milk alternatives has received little attention from scientific research (Verduci et al., 2019). The lack of such information is a critical unmet need, because, these milk alternatives have the potential to contribute to food security, health, and nutrition of a population (Muehlhoff, Bennet, McMahon, & Food and Agriculture Organisation of the United Nations [FAO], 2014). Though dairy alternative beverages are popularly advertised as healthy and wholesome, little research has been done in understanding the consumers' evaluation on and willingness to pay for the main nutritional components. The central question addressed in this article pertains to whether qualitative attributes especially macronutrients and micronutrients have significant effects on consumers' willingness to pay for dairy alternative beverages, what are consumers' subjective evaluations on these attributes and to what extent these attributes influence prices of the products.

As for nutritional contents in the products play an important role in making consumption choices, hedonic pricing model estimation can provide information about consumer's cognition and preference on each qualitative attribute of agricultural products and their willingness to pay for each attribute. Consumers' willingness to pay for purchasing the products (essentially the combination of different qualitative characteristics) and the satisfaction they received from consumption are greatly related to the companies' marketing strategy and selling behavior. Based on the assumption that consumers' utility is gained through consuming the intrinsic properties of a particular good rather than the simple quality and that intrinsic characteristics are combined to constitute the product's market price, this paper contributes to provide manufacturers information about what and how qualitative advancement and differentiation can be made to produce and market better dairy alternative beverages to cater to consumers' preferences. This is of great help to enhance market competitiveness and to expand market share of dairy alternative beverage companies. Also, the results of the study would have important implications for the targeting of nutrition education programs.

## 2 LITERATURE REVIEW

Some existing literature about the nutritional components of dairy alternative beverages are mainly review articles. For example, Vanga and Raghavan (2018) outlined the differences of nutritional contents among various dairy alternative milks (including almond milk, soy milk, rice milk) and cow's milk and through comparison noted that nutritionally soy milk is the best alternative for replacing cow's milk in human diet. Sethi, Tyagi, and Anurag (2016) introduced the functional components of diary alternative beverages and their health benefit of different products appeared in the market and the technological interventions that should be made to improve the quality and acceptability of plant‐based milk alternatives. Mäkinen et al. (2016) gave an overview on the technology of production, nutritional properties, consumer acceptance and environmental impacts of dairy alternative beverages. Verduci et al. (2019) reviewed the different compositions in terms of macronutrients and micronutrients of milk from different mammalian species, including special milk formulas indicated for cow's milk allergy, and of dairy alternative beverages. The empirical research about consumer preference on dairy alternative products embraces Laassal and Kallas (2019) who applied revealed preference discrete choice experiment to analyze consumers' preferences toward dairy‐alternative products in Catalonia using Home‐Scan data of 343 households and the results showed that price was the major driving factor, followed by the original non‐dairy beverage flavor attribute. With the elevated demand on plant‐based alternative milk beverages in U.S., Dharmasena and Capps (2014) estimated the demand for soy milk, white milk, and flavored milk. In addition, Copeland and Dharmasena (2016) analyzed demand for dairy alternative beverages and the effect of increased demand for those products on dairy farmers' welfare.

The concept of food hedonic pricing is first introduced by Waugh (1928) for analyzing the prices of vegetables. He argued that prices of vegetables are closely related to the sizes, lengths, ingredients and other characteristics. Rosen (1974) provided mathematical proof for hedonic pricing model and showed that the intrinsic value of products can be calculated based on econometric methods, thereby analyzing the demand for the bundle of characteristics of certain products. In the year 1966, hedonic pricing models received a great progress. Lancaster (1966) proposed that product attributes (or characteristics) with which the good possessed give rise to utility and not just the quantity of the consumed good. Epple (1987) argued that in the empirical investigation of hedonic models, one issue of interest is to determine how the price of a unit of the commodity varies with the set of characteristics or attributes it possesses. Hedonic approach has been applied in many research areas to measure consumer's willingness to pay for the products. For example, Ghali (2020) applied structural equation modeling to explore the influence of organic food perceived values (utilitarian vs. hedonic) on consumer willingness to buy and willingness to pay for organic oil in a developing country and found that both utilitarian and hedonic values have significant influence on consumer willingness to buy and to pay for organic olive oil. Nepal, Rai, Khadayat, and Somanathan (2020) used hedonic pricing model to analyze the characteristics that affect consumer purchasing decisions on house units in Nepal based on sub‐sample of nationally representative household survey data from urban areas as well as primary data collected from one of the metropolitan cities. Bonanno (2016) used a hedonic price model and 2 years of weekly sales data of yogurts in eight Metropolitan U.S. markets to assess the market value of several health and nonhealth‐related attributes of yogurt, accounting also for their differences across markets. Even though hedonic pricing method has been widely applied in the area of agricultural commodities and other differentiated products, little work has been done to examine the link between the quality attributes and price differentials to explore the pricing mechanism of dairy alternative beverages and conventional milk products. Furthermore, few studies organize and pool the purchase data from Nielsen Homescan in a way that it could not only capture enough qualitative information about the purchased products as well as time effects but also merge with the nutritional data just available from the products' nutrition facts label. Therefore, given the lack of research on dairy alternative beverage market and application of hedonic pricing model to analyze consumers' preference and pricing mechanism of milk alternative beverages, we attempt to (a) develop linear and semi‐log hedonic pricing models for almond milk, soy milk, rice milk and four types of conventional milk (1% fat, 2% fat, fat‐free milk, and whole milk); (b) conduct statistical analysis on all the qualitative characteristics fitted in hedonic pricing models; (c) examine the effect of different characteristics on prices and summarize consumers' preference toward these characteristics.

The organization of the rest of this article is as follows. Section 3 focuses on introducing the methodology applied in this work. We estimate hedonic pricing models, where prices are defined as a function of products' qualitative characteristic. Section 4 focuses on discussing how the data is acquired and organized for this work. Section 5 shows the estimated results of hedonic pricing models. Section 6 offers concluding remarks, research limitations and some interesting future research topics.

## 3 HEDONIC PRICING MODEL DEVELOPMENT

Hedonic pricing models assume that the consumer maximizes utility by selecting products that maximize the sum of the utilities derived from each attribute (Rosen, 1974). Therefore, the price of each beverage in this study can be explained by the set of attributes of the product. *X* = (*X*_1_,*X*_2,_…,*Xj*) represents the qualitative characteristic combination. Qualitative characteristic information has close relationship with prices and hedonic pricing model thus is shown as:

$$P\left(X\right)=P\left(X_{1},X_{2},\ldots ,X_{1}\right)=f\left(x\right)+\varepsilon ,$$

where ε is the error vector, and *P* is the observed price. If the relationship between prices and attributes is assumed to be linear, price of a good *i* can be derived as the sum of the attribute values (Ladd & Suvannunt, 1976). Thus, the total value of each attribute is equal to the quantity of the attribute multiplied by the implicit price of that attribute (Gulseven & Wohlgenant, 2015). The linear and semi‐log hedonic pricing models are constructed as follows:

$$P_{i}=\beta_{0}+\sum_{j}^{}\beta_{j}A_{ij}+\sum_{k}^{}D_{k}X_{ik}+\varepsilon_{i},\;i=1,2,\ldots ,7$$

$$\ln(P_{i})=\beta_{0}+\sum_{j}^{}\beta_{j}A_{ij}+\sum_{k}^{}D_{k}X_{ik}+\varepsilon_{i},\;i=1,2,\ldots ,7$$

where *A_ij_* is the amount of nutritional attribute *j* contained in product *i*. *X_ik_* is other factors that might affect prices. *P_i_* is the monthly average price recorded in Nielsen database by different Universal Product Codes (UPC) that have been purchased from the year 2004 to 2015. These nutritional attributes include calories, fat, fiber, protein, calcium, vitamin A, etc. If the price attribute relationship is assumed to be in a semi‐log form (Nimon & Beghin, 1999), then instead of price, the log‐price of the product is defined regarding attributes as is shown in Equation (3). Similarly, as shown in Equation (2), *P_i_* is the monthly average prices of a beverage from year 2004 to 2015. The implicit prices are the coefficients to be estimated which are represented by *β_j_* and *D_k_*. In the linear hedonic pricing model, the implicit prices or shadow prices can be shown as:

$$\frac{\partial P_{i}}{\partial A_{\mathrm{ij}}}=\frac{\partial f\left(x\right)}{\partial A_{\mathrm{ij}}}=\beta_{j}\;\forall i,j$$

$$\frac{\partial P_{i}}{\partial X_{\mathrm{ik}}}=\frac{\partial f\left(x\right)}{\partial D_{\mathrm{ik}}}=D_{k}\;\forall i,k$$

Marginal effect of semi‐log hedonic pricing model is derived as follows. First, solve for *P_i_* from Equation (3):

$$P_{i}=e^{\left(\beta_{0}+\sum_{j}\beta_{j}A_{\mathrm{ij}}+\sum_{k}D_{k}X_{\mathrm{ik}}+\varepsilon_{i}\right)}$$

then differentiate Equation (5) to get the marginal effect of *A_ij_* and *X_ik_*

$$\frac{\partial P_{i}}{\partial A_{\mathrm{ij}}}=\beta_{j}e^{\left(\beta_{0}+\sum_{j}\beta_{j}A_{\mathrm{ij}}+\sum_{k}D_{k}X_{\mathrm{ik}}+\varepsilon_{i}\right)}=\beta_{j}P_{i}\cdot \forall i,j$$

$$\frac{\partial P_{i}}{\partial X_{\mathrm{ik}}}=D_{k}e^{\left(\beta_{0}+\sum_{j}\beta_{j}A_{\mathrm{ij}}+\sum_{k}D_{k}X_{\mathrm{ik}}+\varepsilon_{i}\right)}=D_{k}P_{i}\cdot \forall i,k$$

Different from previous research, we also take into consideration of time effects on the price of each year by adding yearly dummies into the model. Therefore, all attributes are separated into nutritional attributes and other related attributes that might affect the prices, including package size, values of the multi‐package, brands, coupon, and yearly dummies, etc. *β*_0_ denotes the intercept and $\varepsilon_{i}$ represents the stochastic error term. If we specify all the variables in the model, then the linear and semi‐log hedonic pricing model in this research can be demonstrated as Equations (9) and (10) respectively:

$$\begin{array}{cc} P_{i} & =\beta_{i0}+\beta_{i1}\text{calories}+\beta_{i2}\text{fat}+\beta_{i3}\text{vitamin A}+\beta_{i4}\text{calcium}\\ & \;+\beta_{i5}\text{vitamin D}+\beta_{i6}\text{fiber}+\beta_{i7}\text{protien}+\beta_{i8}\text{brands}\\ & \;+\beta_{i9}\text{coupon}+\beta_{i10}\text{deal}+\sum_{k=1}^{n}\beta_{i1k}{\text{package}}_{\text{size}}\\ & \;+\sum_{m=1}^{n}\beta_{i2m}\text{multi}+\sum_{t=1}^{14}\beta_{i3t}\text{year}+\varepsilon_{i},\;\;\;i=1,2,\ldots ,7 \end{array}$$

$$\begin{array}{cc} \ln P_{i} & =\beta_{i0}+\beta_{i1}\text{calories}+\beta_{i2}\text{fat}+\beta_{i3}\text{vitamin A}+\beta_{i4}\text{calcium}\\ & \;+\beta_{i5}\text{vitamin D}+\beta_{i6}\text{fiber}+\beta_{i7}\text{protien}+\beta_{i8}\text{brands}\\ & \;+\beta_{i9}\text{coupon}+\beta_{i10}\text{deal}+\sum_{k=1}^{n}\beta_{i1k}{\text{package}}_{\text{size}}\\ & \;+\sum_{m=1}^{n}\beta_{i2m}\text{multi}+\sum_{t=1}^{14}\beta_{i3t}\text{year}+\varepsilon_{i},\;\;\;i=1,2,\ldots ,7 \end{array}$$

where $\varepsilon_{i}∼N\left(0,\Sigma^{*}\right)$, Σ* is an *n* × *n* singular covariance matrix. Except for nutritional variables, the hedonic variables include coupon, deal, package size dummies, multi‐pack dummies and yearly dummies, but their values vary for different milk types. *i* represents soy milk, almond milk, rice milk, 2% milk, 1% milk, whole milk and fat‐free milk; *k* is the value of package sizes; *m* is the units purchased together; *t* is regarding to the time series from year 2004 to 2015; *n* means that for different product, their values and numbers of package size and multi‐pack dummies are different as shown in Table 1. From linear and semi‐log hedonic pricing models, we have obtained the marginal value (or shadow price) of each quality attribute available in the product.

**TABLE 1:** Description of dummy variables of package size and multi‐pack

Dummy variables	Soy milk	Almond milk	Rice milk	2% milk	1% milk	Whole milk	Fat‐free milk
*D*_pkge_size1_	8 oz.	8 oz.	11 oz.	size < 8 oz.^b^	8 oz.	size < 8 oz.	size < 8 oz.
*D*_pkge_size2_	8 oz. < size < 10 oz.	10 oz.	12 oz.	8 oz.	10 oz.	8 oz.	8 oz.
*D*_pkge_size3_	10 oz.	12 oz.	14 oz.	8 oz. < size < 10 oz	10 oz. < size < 11 oz	10 oz.	10 oz.
*D*_pkge_size4_	10 oz. < size < 11 oz.	16 oz.	16 oz.	10 oz.	12 oz.	10 oz. < size < 11 oz.	10 oz. < size < 11 oz.
*D*_pkge_size5_	11 oz.	32 oz.	32 oz.	10 oz. < size < 11 oz.	14 oz.	12 oz.	11 oz.
*D*_pkge_size6_	12 oz.	48 oz.^a^	48 oz.	11 oz.	16 oz.	14 oz.	12 oz.
*D*_pkge_size7_	15 oz.	64 oz.	64 oz.	12 oz.	32 oz.	16 oz.	14 oz.
*D*_pkge_size8_	15 oz. < size < 16 oz.		128 oz.^a^	14 oz.	52 oz.	20 oz.	16 oz.
*D*_pkge_size9_	16 oz.			16 oz.	52 oz. < size < 64 oz.	24 oz.	20 oz.
*D*_pkge_size10_	32 oz.			20 oz.	64 oz.	32 oz.	32 oz.
*D*_pkge_size11_	32 oz. < size < 48oz			24 oz.	94 oz.	32 oz. < size < 52 oz.	32 oz. < size < 52 oz.
*D*_pkge_size12_	48 oz.			32 oz.	96 oz.	52 oz.	52 oz.^a^
*D*_pkge_size13_	64 oz.^a^			32 oz. < size < 52 oz.	97 oz.^a^	52 oz. < size < 64 oz.	52 oz. < size < 64 oz.
*D*_pkge_size14_	128 oz.			52 oz.	128 oz.	64 oz.	64 oz.
*D*_pkge_size15_				52 oz. < size < 64 oz. oz.(base)		96 oz.	94 oz.
*D*_pkge_size16_				64 oz.		128 oz.^a^	96 oz.
*D*_pkge_size17_				94 oz.			128 oz.
*D*_pkge_size18_				96 oz.			
*D*_pkge_size19_				97 oz.			
*D*_pkge_size20_				128 oz.			
*D*_multi1_	1^c^ Pack	1 pack	1 pack	1 pack	1 pack	1 pack	1 pack
*D*_multi2_	2 packs	2 packs		2 packs	2 packs	2 packs	2 packs
*D*_multi3_	3 packs			3 packs	3 packs	3 packs	
*D*_multi4_	4 packs	4 packs					
*D*_multi5_						5 packs	
*D*_multi6_	6 packs	6 packs		6 packs	6 packs	6 packs	
*D*_multi7_		12 packs	12 packs	12 packs	12 packs	9 packs	
*D*_multi8_		18 packs					

Note

1. *multi* its value represents number of units in multipack; 2. *multi packs* (i.e. “multi”>1) is total units for a product; 3. *size1_amount* is package size (numeric size of the product).

^a^ The base category of package size dummies and multi‐pack dummies for almond milk, soy milk, rice milk, 2% mill, 1% milk, whole milk and far‐free milk; The base category of multi‐pack dummies for the seven products is *D*_multi1_.

^b^ For package_size dummy variables that are in ranges, for example size1_amount < 8 oz., they are created because there are many package sizes that are not integers and for different types of beverages, the values vary a lot. In order to make the package sizes comparable from one beverage to another, we created some package size dummies that are in ranges.

^c^ 1 is single serve.

## 4 DATA

As shown in Tables 2, the descriptive statistics of variables used in this analysis are listed. The number of variables varies mainly because for different products, their package sizes and multi‐pack values are different. Price variable, as described, is a unit value. Because we also estimate the semi‐log hedonic pricing model, log prices are added. For nutritional variables, the values are based on 8 oz. (1 cup) for each product. The unit of variables including fat, fiber, and protein are grams and that of vitamin A, vitamin D and calcium are percent. It can be shown that the average prices of dairy alternative beverages are generally higher than conventional fluid milk products.

**TABLE 2:** Summary statistics

	Soy milk		Almond milk		Rice milk		2% milk		1% milk		Whole milk		Fat‐free milk	
Variables	Mean	*SD*	Mean	*SD*	Mean	*SD*	Mean	*SD*	Mean	*SD*	Mean	*SD*	Mean	*SD*
P_1_	0.4183	0.2479	0.6369	0.3020	0.4634	0.2557	0.2911	0.1717	0.2399	0.1031	0.3119	0.1773	0.2725	0.1486
ln(P_1_)	−0.9638	0.3849	−0.5225	0.3566	−0.8698	0.4206	−1.3313	0.4257	−1.4991	0.3698	−1.2669	0.4377	−1.3914	0.4119
*x*_Kcal_	105.5549	30.2654	77.4778	35.5234	136.6468	21.7658	129.7193	8.8784	110.7803	4.9886	149.7963	4.3359	84.1034	5.6029
*x*_fat_ (g)	3.3218	1.0906	3.0439	0.6819	2.7584	0.5480	4.9600	0.5683	2.6037	0.5089	7.9789	0.3079	0.0645	0.5950
*x*_fiber_ (g)	1.4017	0.8109	1.4773	1.5467	0.2243	0.9714								
*x*_protein_ (g)	6.6847	1.4877	1.3351	0.9534	1.9499	1.2023	8.1132	0.4813	10.1064	0.7380	8.9530	0.9055	8.2004	0.4014
*x*_VA_ (%)	10.5025	5.8497	14.2548	9.7966	5.5519	3.9113	9.8992	0.6224	30.2281	1.8442	6.0639	0.7108	9.7566	1.4145
*x*_cal_ (%)	32.6198	8.2041	38.0099	11.4905	31.2420	3.0477	29.9992	1.2736	8.0584	0.3556	32.3247	2.4979	30.2431	2.5912
*x*_VD_ (%)	26.7098	9.5451	22.9395	7.6080	13.9236	12.4797	22.9741	6.7407	25.0017	1.3388	24.9657	0.9263	24.5033	3.4929
*D*_deals_	0.2063	0.4047	0.2476	0.4318	0.1502	0.3575	0.1298	0.3361	0.1726	0.3779	0.1150	0.3191	0.1527	0.3597
*D*_brands_	0.2686	0.4433			0.1025	0.3035	0.4268	0.4946	0.5279	0.4992	0.4279	0.4948	0.4645	0.4987
*D*_pkge_size1_	0.0052	0.0721	0.0365	0.1875	0.0024	0.0488	0.0000	0.0029	0.0012	0.0342	0.0000	0.0063	0.0042	0.0646
*D*_pkge_size2_	0.0028	0.0524	0.0038	0.0613	0.0346	0.1828	0.0082	0.0899	0.0005	0.0234	0.0051	0.0710	0.0010	0.0319
*D*_pkge_size3_	0.0145	0.1195	0.0239	0.1527	0.0381	0.1917	0.0009	0.0297	0.0001	0.0110	0.0014	0.0375	0.0002	0.0139
*D*_pkge_size4_	0.0001	0.0096	0.0383	0.1921	0.1120	0.3156	0.0015	0.0389	0.0009	0.0291	0.0016	0.0398	0.0000	0.0067
*D*_pkge_size5_	0.0103	0.1008	0.7454	0.4357	0.1836	0.3873	0.0014	0.0371	0.0003	0.0174	0.0063	0.0792	0.0049	0.0698
*D*_pkge_size6_	0.0055	0.0740	0.0302	0.1711	0.0751	0.2637	0.0002	0.0130	0.0022	0.0473	0.0048	0.0691	0.0005	0.0228
*D*_pkge_size7_	0.0038	0.0612	0.1119	0.3153	0.5304	0.4994	0.0067	0.0816	0.0791	0.2699	0.0717	0.2580	0.0149	0.1211
*D*_pkge_size8_	0.0177	0.1319					0.0024	0.0485	0.0022	0.0469	0.0011	0.0335	0.1265	0.3324
*D*_pkge_size9_	0.0024	0.0488					0.0490	0.2158	0.3813	0.4857	0.0001	0.0114	0.0002	0.0139
*D*_pkge_size10_	0.0920	0.2890					0.0005	0.0220	0.0001	0.0090	0.1575	0.3643	0.0003	0.0165
*D*_pkge_size11_	0.0027	0.0515					0.0001	0.0097	0.0040	0.0633	0.0006	0.0242	0.0003	0.0165
*D*_pkge_size12_	0.0006	0.0253					0.1357	0.3424	0.0026	0.0510	0.0001	0.0109		
*D*_pkge_size13_	0.8400	0.3667					0.0006	0.0253	0.5238	0.4994	0.0009	0.0303	0.0001	0.0106
*D*_pkge_size14_	0.0026	0.0506					0.0001	0.0117			0.3370	0.4727	0.4165	0.4930
*D*_pkge_size15_											0.0045	0.0668	0.0002	0.0135
*D*_pkge_size16_							0.0001	0.0109					0.0065	0.0804
*D*_pkge_size17_							0.3610	0.4803					0.0014	0.0379
*D*_pkge_size18_							0.0001	0.0117						
*D*_pkge_size19_							0.0045	0.0669						
*D*_pkge_size20_							0.0013	0.0353						
*D*_multi1_														
*D*_multi2_	0.0205	0.1415	0.0019	0.0434			0.0091	0.0952	0.0065	0.0806	0.0038	0.0615	0.0085	0.0917
*D*_multi3_	0.0288	0.1672					0.0015	0.0381	0.0051	0.0715	0.0007	0.0266		
*D*_multi4_	0.0003	0.0166	0.0101	0.0998										
*D*_multi5_											0.0003	0.0173		
*D*_multi6_	0.0049	0.0696	0.0082	0.0901			0.0010	0.0318	0.0001	0.0119	0.0000	0.0055		
*D*_multi7_			0.0094	0.0967	0.0358	0.1858	0.0000	0.0029	0.0004	0.0201				
*D*_multi8_			0.0044	0.0662										
*D*_coupon_			0.0830	0.2759	0.0298	0.1701	0.0280	0.1650	0.0401	0.1962	0.0237	0.1522	0.0350	0.1838
*D*_year2014_			0.1157	0.3199	0.0906	0.2872	0.0789	0.2695	0.0507	0.2194	0.0814	0.2735	0.0856	0.2798
*D*_year2013_			0.0924	0.2897	0.0822	0.2749	0.0804	0.2718	0.0519	0.2218	0.0826	0.2753	0.0896	0.2855
*D*_year2012_			0.1025	0.3033	0.1025	0.3035	0.0826	0.2753	0.0534	0.2249	0.0840	0.2774	0.0864	0.2809
*D*_year2011_			0.0949	0.2932	0.0918	0.2889	0.0825	0.2752	0.1227	0.3281	0.1617	0.3682	0.1027	0.3036
*D*_year2010_			0.0874	0.2825	0.0834	0.2767	0.0878	0.2830	0.0658	0.2479	0.0891	0.2849	0.0792	0.2701
*D*_year2009_			0.0767	0.2662	0.0930	0.2906	0.0895	0.2854	0.0018	0.0424	0.0785	0.2690	0.0267	0.1612
*D*_year2008_			0.0685	0.2527	0.0846	0.2785	0.0931	0.2905	0.1305	0.3368	0.1086	0.3111	0.1055	0.3073
*D*_year2007_			0.0572	0.2323	0.0810	0.2731	0.0957	0.2942	0.0734	0.2607	0.0145	0.1194	0.0587	0.2351
*D*_year2006_			0.0459	0.2093	0.0667	0.2497	0.0805	0.2721	0.1679	0.3738	0.0757	0.2645	0.0922	0.2893
*D*_year2005_			0.0264	0.1604	0.0727	0.2598	0.0789	0.2696	0.1159	0.3201	0.0768	0.2663	0.0872	0.2822
*D*_year2004_			0.0308	0.1728	0.0727	0.2598	0.0819	0.2741	0.1155	0.3197	0.0768	0.2663	0.0924	0.2895

**FIGURE 1:**
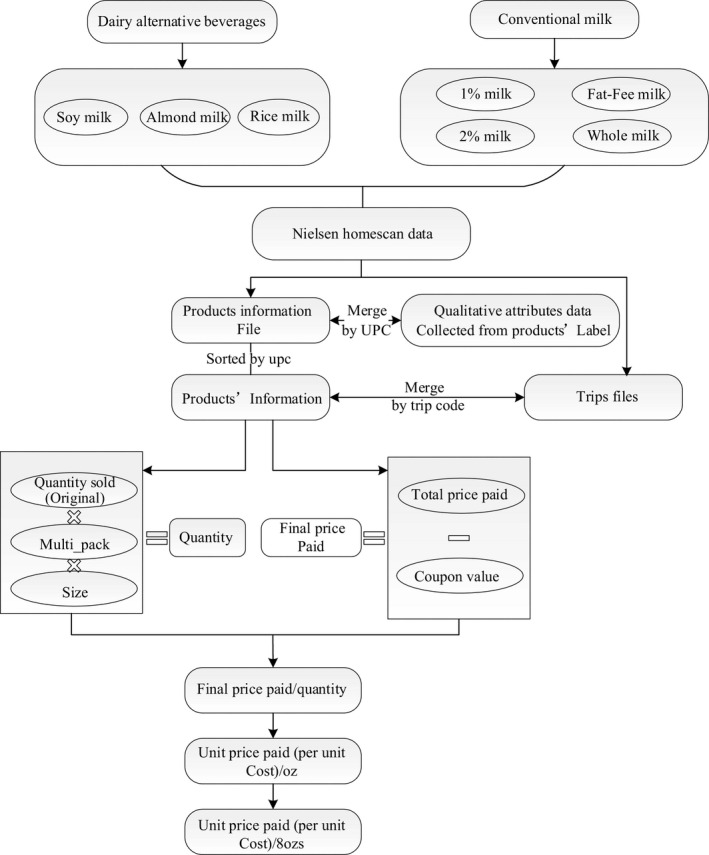
Steps to derive variable‐unit price paid

Three types of dairy alternative beverages (almond milk, soy milk and rice milk) and the four most common types of milk products (whole milk, 1% milk, 2% milk and fat free milk (or skim milk) are included in this work and monthly average price variable is acquired as follows. Monthly average price is the “unit price paid” as shown at the bottom in Figure 1. First, we obtain each product's information from products files; and then we merge the information with trips files to acquire the dataset, which include variables of quantities sold, total price paid by consumers, coupon value, deal_flag_uc, multi_pack, product's package size and size unit.^1^ As Figure 1 shows, the unit price paid (per unit cost) is calculated by first dividing the final price paid by the quantity variable. Final_price_paid is calculated by subtracting the value of variables “coupon value” from the value of “total_price_paid”. Then, we average the unit prices paid in each month in each year to get the monthly average price per oz. and multiply by 8 to get monthly average price per 8oz. (unit monthly average price).

Obtaining data on nutritional information of dairy milk alternative beverages is one of the biggest concerns in estimating the hedonic pricing models due to unavailability of proper database pertaining to such information. In this work, the nutritional variables are obtained from searching for the product label of different products. The USDA Nutritional Database and IRI nutrition information databases are widely used references for most food composition studies. However, even though USDA Nutritional Database includes 50 different categories for varieties of milk products, most of them are conventional milk, with little available information on dairy alternative beverages. Although IRI database has nutritional information of dairy alternative beverages, it uses different UPC system with Nielsen and the nutritional information recorded are not based on uniform unit. Therefore, the only way to obtain nutritional information is gathering the nutritional information directly from the products' label by individual visual observations of beverage packages. The final dataset reflects the same set of qualitative information about characteristics that consumers have based on their products' label. After obtaining the first‐hand data of detailed qualitative characteristics, we merge this with Nielsen based on the products' barcode, or UPC to construct the complete dataset for estimating hedonic pricing models.

Besides the price variable and nutritional variables, we attempt to find out other factors that might exert impact on the prices of those products. The first group of such variables includes package size and multi‐pack.^2^ We also consider that the available deal/coupon when consumers purchase the products should affect prices. Therefore, we select two variables which are deal_flag_uc and coupon_value. “deal_flag_uc” is a dummy variable which indicates if the panelist received a deal. Also if the panelist used a coupon, they enter the amount discounted. If coupon_value and deal_flag_uc are both zero, there is no deal on the purchase. In addition, in order to take into consideration of the time effects on the prices, we add yearly dummies. Since the format of original data file in which households' purchase information is recorded by their trip date, it is very common that the purchase may happen many times each month or no purchase activities within a month. That's one reason why we aggregate the data into UPC level. Another imperative variable considered to affect price is brand. Therefore, a brand dummy is added which equals 1 if it is a store brand and equals zero if it is a national brand.

## 5 EMPIRICAL RESULTS AND DISCUSSION

Applying the model developed in Section 3 and using the data file we constructed in Section 4, we acquired estimates for all the variables considered for each product. The results of linear and semi‐log hedonic regressions are shown in Tables 3 and 4 respectively. In this section, we discuss the empirical results derived from hedonic pricing models in detail and compare and contrast our estimations with those observed in the extant literature.

**TABLE 3:** Estimates of linear hedonic quality attributes

	Soy milk		Almond milk		Rice milk		2% milk		1% milk		Whole milk		Fat‐free milk	
Variables	Estimate	*SE*	Estimate	*SE*	Estimate	*SE*	Estimate	*SE*	Estimate	*SE*	Estimate	*SE*	Estimate	*SE*
Intercept	0.1648***	0.0082	0.2370***	0.0430	0.5850***	0.0734	0.5493***	0.0151	−0.1870***	0.0161	−0.3287***	0.0223	0.1298***	0.0102
*x*_Kcal_	0.0003***	0.0000	0.0003	0.0002	−0.0001	0.0004	−0.0008***	0.0001	0.0016***	0.0001	0.0052***	0.0001	0.0004***	0.0001
*x*_fat_	−0.0025*	0.0010	0.0111	0.0073	−0.1080***	0.0109	0.0050***	0.0008	−0.0124***	0.0009	−0.1133***	0.0019	−0.0016*	0.0007
*x*_VA_	0.0029***	0.0002	0.0073***	0.0007	−0.0140***	0.0020	−0.0137***		0.0188***	0.0005	0.0042***	0.0002	−0.0005***	0.0001
*x*_cal_	0.0008***	0.0001	0.0040***	0.0007	0.0043*	0.0017*	−0.0153***	0.0005	−0.0071***	0.0003	0.0016***	0.0005	0.0012***	0.0001
*x*_VD_	−0.0007***	0.0001	−0.0083***	0.0009	0.0056***	0.0007	0.0024***	0.0001	0.0028***	0.0004	0.0535***	0.0007	−0.0013	0.0010
*x*_fiber_	−0.0065***	0.0014	−0.0082**	0.0035	0.1504***	0.0067								
*x*_protein_	0.0248***	0.0009	0.2115**	0.0074	0.0079	0.0050	0.0383***	0.0011	0.0259***	0.0017	0.0140***	0.0006	0.0051***	0.0003
*D*_brands_	−0.0329***	0.0024			−0.1780***	0.0219	−0.0142***	0.0008	0.4607***	0.0130	−0.0163***	0.0008	−0.0097***	0.0008
*D*_pkge_size1_	0.4837***	0.0174	0.5736***	0.0291	0.2637**	0.0808	4.7131***	0.1284	0.5646***	0.0139	0.2580***	0.0593	0.7955***	0.0058
*D*_pkge_size2_	−0.0127	0.0321	0.4747***	0.0781	0.2635***	0.0330	0.3929***	0.0045	0.6653***	0.0295	0.5022***	0.0054	0.2563***	0.0118
*D*_pkge_size3_	0.7651***	0.0097	0.0644	0.0529	0.1410***	0.0310	0.6927***	0.0127	0.5145***	0.0111	0.1775***	0.0100	0.2685***	0.0269
*D*_pkge_size4_	0.1925*	0.0963	0.6390***	0.0510	0.2418***	0.0282	0.2448***	0.0097	0.5282***	0.0186	0.2333***	0.0105	0.2806***	0.0554
*D*_pkge_size5_	0.7511***	0.0094	0.2877***	0.0470	0.0259	0.0227	0.3801***	0.0101	0.3219***	0.0069	0.4032***	0.0049	0.5783***	0.0054
*D*_pkge_size6_	0.9296***	0.0172			0.1023***	0.0302	0.6159***	0.0287	0.1791***	0.0013	0.3508***	0.0056	0.5051***	0.0164
*D*_pkge_size7_	0.6143***	0.0175	−0.1234***	0.0145	0.0021	0.0244	0.4985***	0.0046	0.0499***	0.0081	0.2344***	0.0019	0.3474***	0.0031
*D*_pkge_size8_	1.0393***	0.0089					0.4586***	0.0078	0.0852***	0.0007	0.2397***	0.0112	0.1781***	0.0012
*D*_pkge_size9_	1.0874***	0.0258					0.3681***	0.0018	0.0754***	0.0360	0.1671***	0.0329	0.0819**	0.0269
*D*_pkge_size10_	0.1648***	0.0036					0.1835***	0.0170	0.2332*	0.0052	0.1141***	0.0014	0.3477***	0.0227
*D*_pkge_size11_	−0.0387*	0.0184					0.7159***	0.0389	0.0577***	0.0064	0.2144***	0.0155	0.3097***	0.0296
*D*_pkge_size12_	0.0507	0.0365					0.1770***	0.0012	−0.1327***	0.0134	0.1412***	0.0343		
*D*_pkge_size13_							0.1197***	0.0149			0.1031***	0.0124	−0.1827***	0.0459
*D*_pkge_size14_	−0.2098***	0.0194					0.2673***	0.0321	−0.1870***	0.0161	0.0480***	0.0010	0.0953***	0.0008
*D*_pkge_size15_											0.0619***	0.0057	0.0688*	0.0278
*D*_pkge_size16_							0.2638***	0.0344					0.1763***	0.0047
*D*_pkge_size17_							0.0949***	0.0009					0.0508***	0.0099
*D*_pkge_size18_							0.0766*	0.0321						
*D*_pkge_size19_							0.1492***	0.0057						
*D*_pkge_size20_							0.0373***	0.0106						
*D*coupon	−0.0226***	0.0045	−0.0431**	0.0147	−0.0916***	0.0196	−0.0208***	0.0025	−0.0146***	0.0019	−0.0204***	0.0027	−0.0148***	0.0023
*D*_multi2_	−0.0410***	0.0069	0.0350	0.0799	0.0479	0.0264	−0.0543***	0.0040	−0.0395***	0.0040	−0.0487***	0.0061	−0.0581***	0.0041
*D*_multi3_	−0.0678***	0.0058					0.0503***	0.0100	0.1009***	0.0046	0.0756***	0.0141		
*D*_multi4_	0.3328***	0.0582	−0.1444***	0.0433										
*D*_multi5_											−0.1771***	0.0241		
*D*_multi6_	0.0787**	0.0269	−0.1336***	0.0389			−0.0245	0.0126	0.3165***	0.0304	−0.5040***	0.0686		
*D*_multi7_			−0.0021	0.0545			−0.2638*	0.1285	0.2274***	0.0207	−0.1304**	0.0451		
*D*_multi8_			−0.1252*	0.0596										
*D*_deals_	−0.0047	0.0028	−0.0310	0.0096	−0.0127	0.0094	−0.0093***	0.0013	−0.0141***	0.0010	−0.0092***	0.0013	−0.0121***	0.0012
*D*_year2014_	−0.0033	0.0043	−0.0203	0.0134	−0.0187	0.0144	0.0113***	0.0020	0.0154***	0.0020	0.0091***	0.0019	0.0185***	0.0018
*D*_year2013_	−0.0138**	0.0043	−0.0083	0.0150	−0.0286	0.0149	−0.0031	0.0020	−0.0060***	0.0020	−0.0070***	0.0019	0.0106***	0.0017
*D*_year2012_	−0.0192***	0.0042	−0.0029	0.0146	−0.0022	0.0142	−0.0145***	0.0019	−0.0107**	0.0020	−0.0101***	0.0019	−0.0032	0.0018
*D*_year2011_	−0.0214***	0.0041	−0.0225	0.0149	−0.0100	0.0147	−0.0134***	0.0019	−0.0051***	0.0017	−0.0142***	0.0017	−0.0057***	0.0017
*D*_year2010_	−0.0157***	0.0041	0.0132**	0.0152	0.0033	0.0158	−0.0411***	0.0019	−0.0323**	0.0019	−0.0408***	0.0019	−0.0284***	0.0018
*D*_year2009_	0.0354***	0.0097	0.0411**	0.0158	−0.0395*	0.0154	−0.0542***	0.0019	−0.0539***	0.0078	−0.0578***	0.0020	−0.0423***	0.0026
*D*_year2008_	0.2175***	0.0165	0.0532	0.0164	−0.0473**	0.0160	−0.0229***	0.0019	−0.0028***	0.0017	−0.0243***	0.0018	−0.0081***	0.0017
*D*_year2007_	−0.0323***	0.0042	0.0211	0.0175	−0.0726***	0.0162	−0.0417***	0.0019	−0.0173	0.0019	−0.0340***	0.0034	−0.0248***	0.0020
*D*_year2006_	−0.0531***	0.0044	0.0074	0.0189	−0.0602***	0.0167	−0.0718***	0.0020	−0.0498***	0.0017	−0.0703***	0.0020	−0.0603***	0.0017
*D*_year2005_	−0.0978***	0.0198	0.0179	0.0233	−0.0756***	0.0165	−0.0701***	0.0020	−0.0520***	0.0017	−0.0660***	0.0020	−0.0609***	0.0018
*D*_year2004_	0.0495	0.0291	−0.0431	0.0221	−0.0885***	0.0163	−0.0748***	0.0020	−0.0545***	0.0017	−0.0709***	0.0020	−0.0643***	0.0017
Sample size	10,904		1,591		839		117,536		49,422		100,390		88,259	
*F* value	1,443.87		204.56		236.16		2,151.21		1,444.51		3,116.00		1906.44	
*P*r > *F*	<0.0001		<0.0001		<0.0001		<0.0001		<0.0001		<0.0001		<0.0001	
RMSE	0.1355		0.1290		0.0846		0.1284		0.0712		0.1185		0.1108	
Adj *R* ^2^	.7987		.8175		.8907		.4402		.5126		.5538		.4441	

Note

*p*‐value = .05 for rejecting the null hypothesis; ***, ** and * indicate significance at 0.001, 0.01, 0.05 levels, respectively.

**TABLE 4:** Estimates of log hedonic quality attributes

	Soy milk		Almond milk		Rice milk		2% milk		1% milk		Whole milk		Fat‐free milk	
Variables	Estimate	*SE*	Estimate	*SE*	Estimate	*SE*	Estimate	*SE*	Estimate	*SE*	Estimate	*SE*	Estimate	*SE*
Intercept	−1.3413***	0.0165	−0.9270***	0.0635	−0.6950***	0.1463	−0.8102***	0.0306	−2.7819***	0.0568	−3.4331***	0.0445	−1.6814***	0.0257
*x*_Kcal_	0.0001	0.0001	−0.0002	0.0003	−0.0018*	0.0007	−0.0022***	0.0001	0.0053***	0.0004	0.0098***	0.0003	0.0025***	0.0002
*x*_fat_	0.0033	0.0021	0.0649***	0.0108	−0.1857***	0.0217	0.0060***	0.0017	−0.0478***	0.0033	−0.1839***	0.0039	−0.0061***	0.0017
*x*_VA_	0.0056***	0.0004	0.0060***	0.0010	−0.0248***	0.0040	−0.0264***	0.0015	0.0589***	0.0017	0.0254***	0.0013	0.0043***	0.0008
*x*_cal_	0.0025***	0.0003	0.0056***	0.0011	0.0098**	0.0033	−0.0428***	0.0010	−0.0210***	0.0011	0.0220***	0.0005	0.0050***	0.0004
*x*_VD_	−0.0011***	0.0003	−0.0136***	0.0013	0.0141***	0.0013	0.0074***	0.0002	0.0046***	0.0014	−0.0045***	0.0009	−0.0005	0.0003
*x*_fiber_	−0.0187***	0.0029	−0.0089	0.0052	0.1401***	0.0133								
*x*_protein_	0.0357***	0.0018	0.1514***	0.0109	0.0136	0.0099	0.1165***	0.0023	0.0799***	0.0060	0.1480***	0.0013	−0.0333***	0.0026
*D*_brands_	−0.0623***	0.0048			−0.3464***	0.0436			1.1251***	0.0457	−0.0384***	0.0016	−0.0264***	0.0021
*D*_pkge_size1_	0.8931***	0.0349	0.7588***	0.0430	0.7094***	0.1610	3.2367***	0.2610	1.3765***	0.0490	0.6397***	0.1182	1.3914***	0.0147
*D*_pkge_size2_	0.1949**	0.0643	0.6859***	0.1154	0.6611***	0.0657	0.9903***	0.0091	1.1757***	0.1043	0.8763***	0.0107	0.8139***	0.0297
*D*_pkge_size3_	1.1565***	0.0194			0.5649***	0.0618	1.5163***	0.0258	1.3205***	0.0393	0.5044***	0.0200	0.8712***	0.0680
*D*_pkge_size4_	0.4572*	0.1926	0.0890	0.0781	0.7195***	0.0563	0.7680***	0.0196	1.1903***	0.0657	0.5959***	0.0210	1.0430***	0.1401
*D*_pkge_size5_	1.1361***	0.0188	0.8575***	0.0753	0.1699***	0.0452	1.0305***	0.0206	0.9907***	0.0242	0.8492***	0.0098	1.3589***	0.0136
*D*_pkge_size6_	1.1577***	0.0345	0.4334***	0.0695	0.4754***	0.0601	1.4012***	0.0584	0.6274***	0.0044	0.7191***	0.0112	1.2257***	0.0414
*D*_pkge_size7_	0.8083***	0.0350			0.1473**	0.0485	1.2354***	0.0095	0.2850***	0.0287	0.5987***	0.0037	1.0189***	0.0079
*D*_pkge_size8_	1.3573***	0.0178					1.1369***	0.0158	0.3515***	0.0025	0.6012***	0.0224	0.6308***	0.0031
*D*_pkge_size9_	1.3775***	0.0515	−0.1833***	0.0214			1.0067***	0.0037	0.3448***	0.1272	0.5192***	0.0656	0.2804***	0.0680
*D*_pkge_size10_	0.3612***	0.0071					0.6408***	0.0346	0.8206**	0.0182	0.3694***	0.0027	0.9848***	0.0573
*D*_pkge_size11_	−0.0823*	0.0368					1.4743***	0.0789	0.2792***	0.0225	0.4528***	0.0308	0.9327***	0.0749
*D*_pkge_size12_	−0.0446	0.0729					0.6081***	0.0024	−1.1616***	0.0473	0.4081***	0.0683	−0.3710**	0.1160
*D*_pkge_size13_							0.2989***	0.0302			0.3606***	0.0247	0.3803***	0.0021
*D*_pkge_size14_	−0.7241***	0.0389					0.8056***	0.0653			0.2053***	0.0020	0.3430***	0.0702
*D*_pkge_size15_									−2.7819***	0.0568	0.2470***	0.0113	0.5997***	0.0120
*D*_pkge_size16_							0.8351***	0.0698					0.2843***	0.0250
*D*_pkge_size17_							0.3689***	0.0017						
*D*_pkge_size18_							0.3371***	0.0653						
*D*_pkge_size19_							0.5269***	0.0115						
*D*_pkge_size20_							0.2012***	0.0216						
*D*_coupon_	−0.0423***	0.0091	−0.0316	0.0217	−0.2683***	0.0391	−0.0964***	0.0052	−0.2333***	0.0142	−0.1081***	0.0055	−0.1043***	0.0058
*D*_multi2_	−0.0603***	0.0137	0.1594	0.1181	0.0651	0.0526	−0.3122***	0.0081	0.3389***	0.0161	−0.2169***	0.0122	−0.2792***	0.0103
*D*_multi3_	−0.1840***	0.0115					0.1550***	0.0204	0.4186***	0.1072	0.1976***	0.0281		
*D*_multi4_	0.2898*	0.1164	−0.1513*	0.0640										
*D*_multi5_											−0.3895***	0.0480		
*D*_multi6_	−0.0393	0.0538	−0.2495***	0.0575			0.0700**	0.0255	0.4233***	0.0732	−0.7621***	0.1369		
*D*_multi7_			−0.0021	0.0545			−0.4437	0.2611	−0.0833***	0.0034	−0.1700	0.0900		
*D*_multi8_			−0.1600	0.0881										
*D*_deals_	−0.0134*	0.0057	−0.0255	0.0142	−0.0347	0.0187	−0.0650***	0.0026	0.0894***	0.0072	−0.0577***	0.0026	−0.0687***	0.0030
*D*_year2014_	−0.0076	0.0086	−0.0040	0.0198	−0.0423	0.0287	0.0566***	0.0040	0.0027	0.0072	0.0509***	0.0038	0.0807***	0.0045
*D*_year2013_	−0.0325***	0.0085	0.0166	0.0221	−0.0631*	0.0297	0.0098*	0.0040	−0.0146*	0.0071	−0.0089*	0.0038	0.0463***	0.0044
*D*_year2012_	−0.0390***	0.0083	0.0200	0.0215	−0.0396	0.0283	−0.0273***	0.0039	0.0060	0.0061	−0.0196***	0.0038	−0.0041	0.0045
*D*_year2011_	−0.0480***	0.0082	−0.0066	0.0221	−0.0423	0.0293	−0.0319***	0.0039	−0.1124***	0.0068	−0.0201***	0.0034	−0.0142***	0.0043
*D*_year2010_	−0.0470***	0.0082	0.0546*	0.0225	0.0106	0.0315	−0.1253***	0.0039	−0.2117***	0.0274	−0.1134***	0.0038	−0.1055***	0.0046
*D*_year2009_	0.0539**	0.0194	0.0766**	0.0234	−0.0831**	0.0308	−0.1783***	0.0039	0.0335***	0.0060	−0.1768***	0.0039	−0.1523***	0.0066
*D*_year2008_	0.3445***	0.0331	0.1107***	0.0242	−0.0942**	0.0319	−0.0367***	0.0038	−0.0412***	0.0066	−0.0273***	0.0036	−0.0020	0.0042
*D*_year2007_	−0.0766***	0.0084	0.0724**	0.0258	−0.1244***	0.0322	−0.1104***	0.0038	−0.1872***	0.0058	−0.1053***	0.0068	−0.0744***	0.0050
*D*_year2006_	−0.1117***	0.0089	0.0443	0.0279	−0.1015**	0.0333	−0.2384***	0.0040	−0.1680***	0.0061	−0.2053***	0.0039	−0.2251***	0.0044
*D*_year2005_	−0.0816*	0.0396	0.0915**	0.0344	−0.1347***	0.0329	−0.2233***	0.0040	−0.1787***	0.0061	−0.1815***	0.0039	−0.2100***	0.0045
*D*_year2004_	−0.0107	0.0582	−0.0128	0.0326	−0.1671***	0.0325	−0.2427***	0.0040	0.0894***	0.0072	−0.2000***	0.0039	−0.2316***	0.0044
Sample size	10,904		1,591		839		117,536		49,422		100,390		88,259	
*F* value	749.46		112.48		151.88		749.46		112.48		4,543.94		2,773.87	
*P*r > *F*	<0.0001		<0.0001		<0.0001		<0.0001		<0.0001		<0.0001		<0.0001	
RMSE	0.1923		0.2002		0.1685		0.1923		0.2002		0.2609		0.2801	
Adj *R* ^2^	.7400		.6849		.8394		.7400		.6849		.6243		.5376	

Note

*p*‐value = .05 for rejecting the null hypothesis; ***, ** and * indicate significance at 0.001, 0.01, 0.05 levels, respectively.

The hedonic results generally conform to our expectations. It is rarely witnessed in the existing literature that applies hedonic pricing method incorporate as many variables as in our research, but both model forms of hedonic regressions we developed still fit well for dairy alternatives and conventional milk data. In terms of model performance, it can be observed form Tables 3 and 4 that *F* test for each hedonic pricing model has *p* < .001 indicating that the model fits the data well; the adjusted *R*‐squared is greater than 0.5 in terms of all semi‐log hedonic pricing models also prove that the models have good fitness; RMSE for each model is small enough (around 0.1) to claim that the both model forms fit well for the data.

Regardless of the functional forms, almost all the nutritional variables are significant with only few exceptions. Compared with soy milk which has all the nutritional variables significant in linear functional form, fat content is not significant for almond milk and protein is not significant for rice milk. There are several possible explanations. First, there might have some confounding variable we do not consider in the models and thus affecting the significance of some coefficients; second, rice milk is launched much later than soy milk and almond milk in the market, thus by the year 2015 there are not enough purchase data for rice milk in Nielsen Homescan database. Also rice milk product categories and brands are significantly less than soy milk and almond milk. The lack of product and purchase information and thus restricted samples might cause insignificant effect of protein on prices.

As indicated by the intrinsic meaning of hedonic pricing model, the estimated coefficient for each characteristic variable shows consumers' willingness to pay for the specified characteristic keeping other variables constant, thus being a reflection of consumers preference toward it. As intuition suggests, if consumers' acceptance of a product is discouraged due to the presence of a given attribute, their willingness to pay for that attribute should be negative; as a result, the implicit price attached to such attribute may be negligible, or even negative. The estimated coefficient $\beta_{j}$ in linear hedonic model indicates the contribution of unit increase in the nutrient to the change of unit price on average while the estimated coefficient $D_{k}$ captures the change of log unit price caused by unit increase in each attribute.

In terms of the effect of fat content, Gulseven and Wohlgenant (2015) found that lipid fat contributes 0.861 to prices of milk products considered in their study. Comparatively, in linear hedonic pricing models, fat content contributes negatively to unit price (monthly average price per 8 oz.) in our study with estimated coefficient −0.1080 for rice milk and −0.0025 for soy milk, reflecting that if fat content is raised by one unit, consumers' willingness to pay for unit price decrease of rice milk and soy milk are on an average 10.8 percent and 0.25 percent respectively. However, fat contributes positively to prices of almond milk in semi‐log hedonic pricing model. The contrasting result is possibly caused by model specifications and product categories taken into account. The research results of Gulseven and Wohlgenant (2015) are based on a composite viewpoint by which they aggregate several milk products and soy milk in to one product type, while our work examines different product separately. In addition, one likely reason for consumers' willingness to pay for fat content is that fat contributes to the texture, flavor, and aroma of a wide variety of foods (Drewnowski & Almiron‐Roig, 2010), the consumers who have a strong preference for flavor and taste of food might be willing to pay more for fat.

Instead of considering calorie content, Gulseven and Wohlgenant (2015) include carbohydrate content in their model and the estimation results show that carb content exerts positive effect on prices. Similarly, calorie has significant positive effect (0.03%) on unit prices of soy milk but has negative effect on unit prices of rice milk (−0.01%) in linear hedonic model. It is commonly known that food provides energy to the body in the form of calories and the energy in dairy alternative beverages comes from protein, carbohydrate and fat content. As suggested by Drewnowski (2015), Americans were advised to get the most nutrition out of their calories and to make smart, nutrient‐dense choices from every food group. Taubes (2008) argued that there are good and bad calories; the key to good health is the kind of calories we take in, not the number. As shown in Table 3, vitamin A contributes positively to prices of soy milk and almond milk, with coefficient being 0.0029 and 0.073 respectively, but it contributes negatively to rice milk. It is interesting to find that vitamin D, however, has the mirror effect on these three beverage types. These results can be possibly explained by consumers different attitudes toward these two vitamins. By analogy with Bonanno (2016)'s study which shows that fiber, a health‐related attribute in food products, is perceive unfavorably by yogurt consumers if the yogurt is enriched and fortified with fiber, even though vitamin A and vitamin D are considered as a beneficial nutrient, consumers might have negative attitude toward them when they are artificially enriched in dairy alternative beverages. Also, Willett (2013) indicates that when it comes to vitamins and minerals, the notion of “the more, the better” is incorrect since nutrients can be harmful when taken in amounts above what is considered beneficial and multivitamin is one of them. The positive and significant effect of protein and calcium on prices manifest consumers' favorable acceptance of these nutritional attributes. Comparing different weights of nutritional variable from Tables 3 and 4, we can witness that among all the nutritional variables considered, protein has highest weight meaning it is regarded by consumers as the most preferred qualitative characteristics for soy milk and almond milk and calorie is least valued by consumers.

The dummy variable “Brands” has negative sign and is significant as expected, indicating that prices of private label products are lower than that of national brand products. Packaging size and shape are also significant factors in designing the package and a decision‐making instrument (Ksenia, 2013). We can witness that in our study almost all package‐size dummy variables have positive and significant contribution to prices and this offers essential information for the companies about the consumer attraction and importance of designing attributes. In general, people prefer smaller sizes at least not bigger than 64 oz. per package. Coupons and deals have negative and significant effects on unit price. Similar results can be found in Gulseven and Wohlgenant (2015)'s study in which the estimated coefficient of marketing promotion dummy is −1.583. The effects of multi‐pack dummies have little inconsistency. Regarding to soy milk, pack of 4 and 6 have positive influences meaning that these two package units are preferred than pack of 1, but for almond milk and rice milk, consumers are inclined to purchase pack of 2 rather than other multi packages. This result is in line with works of Bonanno, Bimbo, Costanigro, Lansink, and Viscecchia (2019) and Bimbo, Bonanno, and Viscecchia (2016) showing that presence of a two‐compartment package (Two Compartments) show positive implicit prices of Italian yogurt. The package preference should be taken seriously by manufacturers to make optimal production policy. Yearly dummies are significant for soy milk and rice milk, with year 2004 to year 2009 showing the greatest significance, but the same significant effects are not observed on almond milk except for year 2008 and 2009.

Comparatively, in semi‐log model, all package sizes dummies have positive and significant effects on unit price. The impact of multi‐pack dummies is almost the same as it in the linear hedonic form too. Also, the yearly dummies do not show much significance for almond milk except for year 2008 in which a great increase about 11% on unit price is observed. In term of 2% and 1% reduced fat milk, almost all the variables are significant at 0.1% level. One interesting result is that calcium has negative effect on prices of reduced fat milk but has positive effects on prices of other milk products. But in general, consumers treat the acquisition of calcium as important component of a healthy diet.

## 6 CONCLUSIONS, LIMITATIONS AND FUTURE RESEARCH

In terms of rate of growth, dairy alternative beverage market in the United States has surpassed the growth of conventional milk market in recent years. The ongoing competition between dairy alternative beverages and conventional milk is expected to intensify over the next several years as consumers become more comfortable with milk alternative beverages and criticism of dairy foods continues to grow. This work focuses on analyzing the relationship between the qualitative characteristics embedded in the differentiated dairy alternative beverages and their market prices. We take product characteristics approach, specifically hedonic pricing model to study consumers' preference and their willingness to pay for the qualitative characteristics. By investigating the existing research about hedonic pricing model in different agricultural area and comprehensively taking into consideration of factors that might affect prices of dairy alternative beverages and conventional milk, we constructed a valid model to explain and estimate prices of these products. The price composition of these products is directly related to multiple factors which include not only the qualitative attributes, such as nutritional content, brand, package size and multi packages but also such common factors as supply and demand. This work focuses mainly on how the nutritional attributes and other qualitative characteristics affect the formation of price mechanism. The estimation results indicate that both linear and semi‐log hedonic pricing models fit the data of seven products very well. Regardless of the functional forms of hedonic pricing models, almost all the nutritional variables have significant effects on prices implying that nutritional contents are seriously considered when consumers make purchasing decisions. Health‐related nutritional attributes such as protein and calcium are widely recognized by consumers and certain amount of good fat and calories are also accepted. Protein is the most valued attribute by consumers. The multivitamins, Vitamin A and D, should be cautiously considered by manufactures since consumers feel more encouraged to purchase dairy alternative beverages contained with natural nutritional attributes. The estimation results also suggest that selecting appropriate qualitative attributes to tap into the demand of dairy alternative beverages can result in successfully differentiated products.

This analysis does however show limitations. Due to data limitations, we can only use pooled UPC level information to estimate the hedonic pricing models. Because milk alternative beverages are starting to gain ground in the recent years, adequate purchase observations were not available at the beginning of the time period pertaining to this study. In addition, we need variations on the nutritional attributes, but household level data cannot guarantee enough variability. Therefore, we consider pooled data which can not only capture variability of the nutritional attributes but also enable us to expand the time period to be considered. Data limitations have also constrained our selection of related dairy alternative beverages from which we can only include soy milk, almond milk and rice milk. Besides, the information about nutritional data is very scarce and limited for dairy alternative beverages in the Nielsen Homescan database and also in the USDA nutritional database. Therefore, bulk of the nutritional data was collected from product labels. The estimated results of demand could possibly be more definitive and convincing if data after the year 2010 were used and more nutritional information about diary alternative beverages is available. Another limitation is that the analysis performed cannot provide insights on the impacts of demographic variation across markets.

Studying consumer behavior cannot leave without consumer demand analysis. Traditionally, consumer demand is analyzed using demand system such as AIDS, Rotterdam and some modifications to these two models. The basic assumption for these conventional demand models is that consumers' utility is obtained from the quantity of goods they consumed which is also the assumption of hedonic pricing model as aforementioned. However, the conventional demand system estimation is complicated in that the number of parameters we need to estimate is large. Therefore, some innovative method to estimate demand which is based on hedonic pricing model estimation and then reparameterizing the estimators is developed such as Distance Matrix method and Hedonic Metric approach. The latter is based on hedonic pricing model estimates to estimate demand of milk products. This approach overcomes the shortages of Distance Matrix method but also greatly reduced the number of parameters to be estimated. It is expected that our future work will develop this method and apply it into the analysis of demand on dairy alternative beverages to explore their expenditure, own‐price and cross‐price elasticities.

## CONFLICT OF INTEREST

We declare that we do not have any conflict of interest.

## ETHICAL REVIEW

This study does not involve any human or animal testing.

## INFORMED CONSENT

Written informed consent was obtained from all study participants.

## ENDNOTES

^1^ size1_units is the unit of measure. For example, “size1_amount” might be “16.0”, and “ size1_units” might be “OZ.”; coupon is total discount for amount due to coupon; deal_flag_uc is presence of a deal (1 = deal, 0 = no deal).

^2^ These variable names used in this study are referenced to the original variable names in Neilson HomeScan dataset.
